# Robustly Adaptive EKF PDR/UWB Integrated Navigation Based on Additional Heading Constraint

**DOI:** 10.3390/s21134390

**Published:** 2021-06-26

**Authors:** Debao Yuan, Jian Zhang, Jian Wang, Ximin Cui, Fei Liu, Yalei Zhang

**Affiliations:** 1 School of Geoscience and Surveying Engineering, China University of Mining and Technology-Beijing (CUMTB), Beijing 100083, China; yuandb@cumtb.edu.cn (D.Y.); cxm@cumtb.edu.cn (X.C.); 2 School of Geomatics and Urban Spatial Informatics, Beijing University of Civil Engineering and Architecture (BUCEA), Beijing 102616, China; wangjian@bucea.edu.cn (J.W.); pntrc@cumt.edu.cn (F.L.); zhangyalei_hdxy@163.com (Y.Z.)

**Keywords:** indoor positioning, PDR/UWB, adaptively robust EKF, loose combination, heading constraints

## Abstract

At present, GNSS (Global Navigation Satellite System) positioning technology is widely used for outdoor positioning services because of its high-precision positioning characteristics. However, in indoor environments, effective position information cannot be provided, because of the signals being obscured. In order to improve the accuracy and continuity of indoor positioning systems, in this paper, we propose a PDR/UWB (Pedestrian Dead Reckoning and Ultra Wide Band) integrated navigation algorithm based on an adaptively robust EKF (Extended Kalman Filter) to address the problem of error accumulation in the PDR algorithm and gross errors in the location results of the UWB in non-line-of-sight scenarios. First, the basic principles of UWB and PDR location algorithms are given. Then, we propose a loose combination of the PDR and UWB algorithms by using the adaptively robust EKF. By using the robust factor to adjust the weight of the observation value to resist the influence of the gross error, and by adjusting the variance of the system adaptively according to the positioning scene, the algorithm can improve the robustness and heading factor of the PDR algorithm, which is constrained by indoor maps. Finally, the effectiveness of the algorithm is verified by the measured data. The experimental results showed that the algorithm can not only reduce the accumulation of PDR errors, but can also resist the influence of gross location errors under non-line-of-sight UWB scenarios.

## 1. Introduction

At present, with the rapid development of mobile communication technology, people are paying increasing attention to location-based services. Statistics show that 80% of location-based services are indoors [1], so it is necessary to establish a high-precision and continuous indoor positioning system. In recent years, wireless positioning technology for indoor environments has been widely studied, including pseudolite technology, Bluetooth, infrared, Ultra-Wide Band (UWB), and Pedestrian Dead Reckoning (PDR). Of course, with the development of technology, some new positioning methods have also been adopted (such as occupancy grid maps [2,3]). In order to further improve the reliability and accuracy of indoor positioning, multi-sensor fusion positioning technology has attracted the attention of scholars at home and abroad; the Extended Kalman Filter (EKF) algorithm is widely used in multi-sensor fusion positioning systems. However, the complexity of indoor environments makes it difficult to build high-precision dynamic models and observation models effectively.

In recent years, UWB positioning technology has developed rapidly. Compared with Bluetooth, pseudolite, and other technologies, UWB can penetrate walls of a certain thickness, meaning that it has a strong anti-multipath ability, high positioning accuracy, and is conducive to providing real-time positioning services in sheltered and complex environments [4]. At present, the common UWB positioning methods are as follows: (1) One-Way TOA Ranging; (2) Two-Way TOA Ranging; (3) Time Difference of Arrival [5]. However, due to the complexity of indoor environments, UWB signals are often received without line-of-sight, in addition to other factors influencing the process of transmission, resulting in large deviations in the positioning results [6]. In order to reduce the influence of the gross error of non-line-of-sight (NLOS) locations in the UWB, neural network, support vector machine, Gaussian process regression, and other algorithms are used to identify the NLOS range value of UWB [7,8]. Yang proposed a NLOS error elimination method based on a sparse pseudo-input Gaussian process, which has a low complexity and offers high performance in NLOS scenes [9]. Ridolfi proposed the use of the combination of a CNN neural network algorithm and adaptive error correction algorithm to eliminate NLOS errors and improve the positioning accuracy of the base station [10]. Liu reduced the influence of NLOS errors based on the SVM algorithm under the condition of considering various error factors, such as transmission distance of the signal, the transmitted power, and the transmitted preamble length [11]. Cui combined the Morlet wavelet transform with a CNN neural network algorithm to extract the observed values with a non-horizon error. The experimental results showed that the algorithm is more accurate [12]. PDR positioning technology uses acceleration sensors and gyroscopes to detect the step frequency, estimate the step length, and calculate the heading, and then to calculate pedestrian positions; it offers high positioning continuity and high positioning accuracy over a short time period [13]. However, there is a serious error accumulation problem with PDR positioning technology. Cho et al. [14] proposed using a neural network for step size estimation, which can effectively avoid the interference of accelerometer bias in the step size estimation. As one of the important parameters of PDR, the accuracy of the course estimation determines the positioning performance. Klingbeil et al. [15] achieved an accurate heading correction by combining the magnetometer and gyroscope measurement data. At present, the mainstream heading correction method involves the use of short-baseline wireless sensor technologies to correct the PDR positioning system, including Bluetooth [16], UWB [17], and wireless fidelity [18].

At present, the reliance on single-sensor indoor positioning algorithms makes it difficult to meet the demands of high-precision location services in complex indoor environments. Multi-sensor combined positioning algorithms provide development potential for high-precision indoor positioning. To further improve the accuracy of PDR indoor pedestrian dynamic positioning, an unscented Kalman filter was used for PDR/UWB fusion positioning. The experimental results showed that the dynamic and static positioning accuracy of the fusion algorithm can be controlled at the decimeter level in open areas; however, in long and narrow indoor environments (such as corridors), the network structure of the UWB base station is poor and the solution equation appears to display the rank deficiency phenomenon, which will reduce the accuracy and availability of the PDR/UWB positioning algorithm [19]. Wang et al. [20] achieved WiFi–P-O fusion positioning based on a fading adaptive Kalman filter by using a multi-sensor device from a smart phone. The algorithm achieved accurate correction of inertial navigation unit heading information by adding map constraints. However, because the accuracy of the algorithm mainly depends on WiFi positioning results, its positioning performance cannot meet the requirements of high-precision positioning. For geomagnetic navigation, the integrated WiFi–geomagnetic matching algorithm can effectively reduce the search range of the geomagnetic matching algorithm, so as to improve the matching success rate [15]. Li et al. [21] used an EKF to achieve PDR/UWB fusion positioning, which can resist the influence of gross errors in UWB data to a certain extent. However, because the algorithm only takes the UWB ranging value as the observation, once the UWB data are missing, the positioning accuracy of the algorithm will be difficult to control. Corrales et al. [22] used a particle filter (PF) algorithm for UWB, IMU, and odometer fusion positioning. The experimental results showed that the algorithm can obtain high-precision positioning data in non-line-of-sight environments. However, compared with EKF, the PF algorithm has a complex process and low computational efficiency.

Robustly adaptive filters can effectively resist the influence of gross errors between a dynamic model and an observation model. The adaptive Kalman filter algorithm can resist the influence of gross errors on dynamic models, while the robust Kalman filter algorithm can resist the influence of gross errors on the observation values. Yang et al. [23,24,25,26,27] carried out a dynamic geodesy approach based on a robustly adaptive Kalman filter. This method can not only effectively balance the contribution rate of the dynamic and observation models for the optimal estimation of parameters, but can also resist the influence of gross errors on the positioning results to a certain extent. In the past few years, the Kalman filter has been widely used in various fields, such as in INS dynamic alignment [28,29,30], the INS/SAR fusion positioning model [31], real-time clock error estimation [32], precise single-point positioning [33], satellite orbit determination [34], radar navigation [35], and SLAM [36]. Li et al. [37] implemented WiFi/PDR fusion positioning based on a robustly adaptive Kalman filter. The experimental results showed that the algorithm can effectively resist the influence of gross errors by controlling the system variance and observation noise in different scenes. Liu et al. [38] loosely combined UWB and PDR based on the EKF algorithm and ensured PDR positioning accuracy through map heading constraints. The experimental results showed that the location result for the UWB/PDR fusion with map constraints was best. However, the influence of dynamic model errors was not considered in this algorithm. Guo et al. constructed a NLOS error recognition method based on the intensity distribution characteristics of the UWB signal, and they realized the combined location of PDR and UWB based on the Kalman filter [39]. Tong et al. realized the combined location of PDR and UWB based on the weighted method, but the best way to determine the weight factor for different scenes remains to be studied [40].

Aiming to mitigate PDR error accumulation and non-line-of-sight (NLOS) positioning gross errors in UWB, a PDR/UWB positioning algorithm based on an adaptively robust Kalman filter is proposed in this paper. The algorithm not only adaptively adjusts the variance of the dynamic model to resist the gross error of the dynamic model according to the positioning scene, but also adaptively adjusts the random model of the observed value by using the robust factor. At the same time, the algorithm dynamically adjusts the initial random model according to the type of observation to improve the optimal estimation accuracy of the PDR–UWB positioning model. In order to further improve the accuracy of the model, the algorithm determines the PDR heading constraint mode according to the positioning scene.

The article is divided into four sections. Section 2 introduces the UWB positioning principles, the PDR positioning principles, the adaptively robust Kalman filter algorithm, and the fusion positioning model with additional heading constraints. Section 3 uses the measured data to demonstrate and analyze the algorithm, and Section 4 analyzes and summarizes the algorithm.

## 2. Methods

### 2.1. UWB Positioning System

For indoor positioning based on the UWB system, the location of a mobile station is determined by measuring the real-time distance between the mobile station and a fixed reference station. In this paper, the basic Kalman filtering algorithm is used to solve the UWB positioning. The position and speed of the mobile station are taken as the state parameters $X_{k}={\left[\begin{array}{cccc} x_{k} & y_{k} & z_{k} & \begin{array}{cc} v_{x,k} & v_{y,k}\begin{array}{l} v_{z,k} \end{array} \end{array} \end{array}\right]}^{T}$, where $x_{k}$, $y_{k}$, $z_{k}$, $v_{x,k}$, $v_{y,k}$, and $v_{z,k}$ represent the coordinate values and speed of the mobile station at time k; the dynamic model of the UWB positioning system is as follows:

(1) $$X_{k}=FX_{k-1}+w_{k}$$

where $X_{k}$ and $X_{k-1}$ represent the state vectors at times k and k−1, F is the state transition matrix (Formula (2), where t represents the sampling interval), $w_{k}$ is the system noise vector, and its covariance matrix is $Q_{k}$.

(2) $$F=\left[\begin{array}{llllll} 1 & 0 & 0 & t & 0 & 0\\ 0 & 1 & 0 & 0 & t & 0\\ 0 & 0 & 1 & 0 & 0 & t\\ 0 & 0 & 0 & 1 & 0 & 0\\ 0 & 0 & 0 & 0 & 1 & 0\\ 0 & 0 & 0 & 0 & 0 & 1 \end{array}\right]$$

The TOA ranging model [1] is used for UWB ranging. The distance between the UWB base station and the mobile station is obtained by measuring the time difference between the UWB signal from the base station and the tag. The ranging model from the mobile station to the base station at time k is as follows:

(3) $$d_{r,k}^{i}=\sqrt{{\left(x_{r}-x^{i}\right)}^{2}+{\left(y_{r}-y^{i}\right)}^{2}+{\left(z_{r}-z^{i}\right)}^{2}}=c(t_{r}-t^{i})$$

where $d_{r,k}^{i}$ is the ranging value; $x_{r}$, $y_{r}$, and $z_{r}$ are the mobile station coordinate values; $x^{i}$, $y^{i}$, and $z^{i}$ are the base station coordinates; $t_{r}$ is the UWB signal receiving time; $t^{i}$ is the signal transmitting time; c is the speed of light.

According to Equation (3), the measurement equation for the UWB positioning system is as follows:

(4) $$Z_{k}=f(X_{k|k-1})+V_{k}$$

where $Z_{k}$ represents the observation vector composed of the ranging value, $X_{k|k-1}$ is the system state prediction value, $f(X_{k|k-1})$ is the nonlinear expression of Formula (3), and $V_{k}$ is the residual vector. Taylor expansion is carried out for Formula (3) to obtain the linear coefficient matrix $H_{k}$:

(5) $$H_{k}=\left[\begin{array}{cccccc} \frac{x_{r}-x^{1}}{\rho_{r}^{1}} & \frac{y_{r}-y^{1}}{\rho_{r}^{1}} & \frac{z_{r}-z^{1}}{\rho_{r}^{1}} & 0 & 0 & 0\\ \frac{x_{r}-x^{2}}{\rho_{r}^{2}} & \frac{y_{r}-y^{2}}{\rho_{r}^{2}} & \frac{z_{r}-z^{2}}{\rho_{r}^{2}} & 0 & 0 & 0\\ \vdots & \vdots & \vdots & \vdots & \vdots & \vdots \\ \frac{x_{r}-x^{i}}{\rho_{r}^{i}} & \frac{x_{r}-x^{i}}{\rho_{r}^{i}} & \frac{z_{r}-z^{i}}{\rho_{r}^{i}} & 0 & 0 & 0 \end{array}\right]$$

where $\rho_{r}^{i}$ is the calculated value of the distance from the mobile station r to the base station i according to the predicted value of the system state.

**Figure 1:**
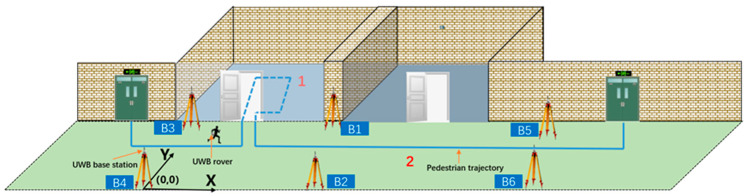
Model of the UWB (Ultra Wide Band) indoor positioning system.

Figure 1 shows the indoor pedestrian positioning system based on UWB. First, the UWB base stations are evenly distributed along the corridor to construct the local coordinate system. Then, the pedestrian carries the UWB mobile station indoors and gives the pedestrian’s position in real time based on the above UWB positioning model. The indoor activity trajectory of the pedestrians is divided into two parts: position 1 for pedestrians entering the room and position 2 for pedestrians in the corridor. When the pedestrian is at position 1, due to the influence of gross errors (such as NLOS errors), the positioning results will involve serious deviation.

### 2.2. PDR Positioning System

The PDR location algorithm is used to track the spatial position of a pedestrian’s trajectory. Its principal aim is to obtain the heading angle by using the equipment direction sensor, to calculate the user’s step value and step speed by using the acceleration sensor, and finally to calculate the location information of the mobile terminal, which has the characteristics of short-time positioning accuracy and strong independence.

#### 2.2.1. Gait Detection with Multi-Threshold Constraints

The accuracy of the step size estimation directly affects the positioning accuracy, and its value can be obtained simultaneously with gait recognition. Therefore, accurate gait detection is the basis of step size estimation. Multi-threshold constrained gait detection uses the numerical acceleration threshold and time threshold for gait detection [20]. Taking peak detection as an example, its principles are as follows.

Let $Num_{pk}$ and $Num_{vy}$, respectively, represent the detected peak and valley number. If only one peak value is detected ($Num_{pk}-Num_{vy}=1$), the expression for peak detection is

(6) $$flag=\{\begin{array}{l} 1,\delta_{\Delta t_{pv}}=\frac{1}{2}\delta_{\Delta t_{p}},a_{p}\geq \delta_{\Delta_{p}}\&\Delta t_{pv}\geq \delta_{\Delta t_{pv}}\&\Delta t_{p}\geq \delta_{\Delta t_{p}}\\ 0,\delta_{\Delta t_{pv}}=\frac{1}{2}\delta_{\Delta t_{p}},others \end{array}$$

When two continuous peaks are detected ($Num_{pk}-Num_{vy}=2$), the peak detection expression is as follows:

(7) $$flag=\{\begin{array}{l} 1,\delta_{\Delta t_{pv}}=\frac{1}{2}\delta_{\Delta t_{p}},a_{p}\geq \delta_{\Delta_{p}}\&\Delta t_{pv}\geq \delta_{\Delta t_{pv}}\&\Delta t_{p}\geq \delta_{\Delta t_{p}}\&\Delta a_{p}>0\\ 0,\delta_{\Delta t_{pv}}=\frac{1}{2}\delta_{\Delta t_{p}},others \end{array}$$

where $\delta_{\Delta t_{pv}}$ is the time difference threshold between adjacent peaks and troughs, $\delta_{\Delta t_{p}}$ is the time difference threshold between adjacent peaks, $\delta_{\Delta_{p}}$ is the acceleration peak threshold, $\Delta a_{p}$ is the difference between adjacent peaks, $\Delta t_{pv}$ is the time difference between adjacent peaks and troughs, $\Delta t_{p}$ is the difference between adjacent peaks, $a_{p}$ is the acceleration measurement value to be verified, and flag is used to indicate whether the point is a peak (1: yes, 0: no).

In order to ensure the correctness and adaptability of the acceleration peak threshold, the dynamic threshold method is adopted:

(8) $$\delta_{\Delta_{p}}=\{\begin{array}{l} \frac{1}{\Delta t}\int_{t_{k-1}}^{t_{k}}a(t)dt,k=2\\ 1,k=1 \end{array}$$

where $t_{k-1}$ and $t_{k}$, respectively, represent the observation times for the beginning and end of k−1 and k, and a(t) is the real-time measurement value of the accelerometer.

**Figure 2:**
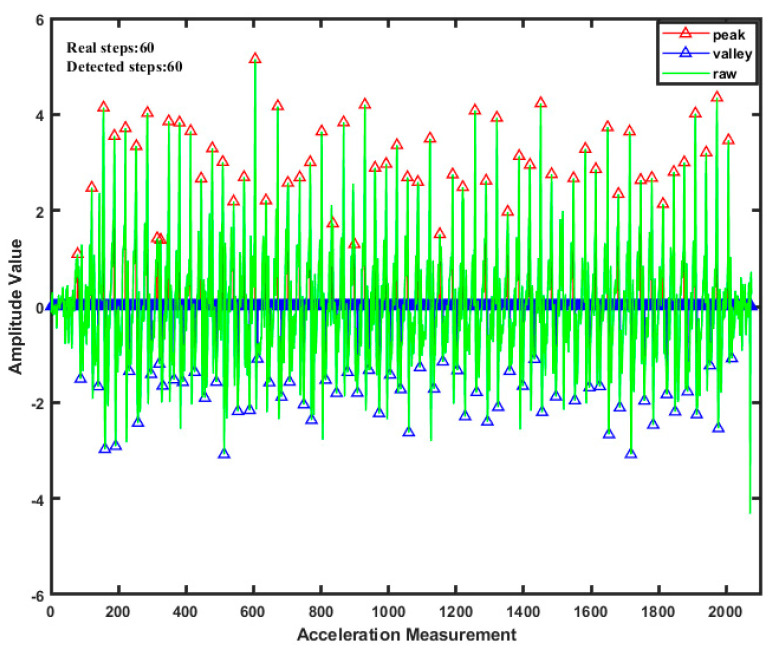
PDR (Pedestrian Dead Reckoning) gait detection.

Figure 2 shows a set of experiments using the above algorithm to detect the gait of the PDR. The experimental results show that the pedestrian’s steps can be accurately detected by setting a multi-threshold.

After the pedestrian’s gait is accurately detected, the step estimation can be obtained using the following formula:

(9) $$L_{k}=K\sqrt[{4}]{a_{\max}-a_{\min}}$$

where $L_{k}$ is the step size at the k step; $a_{\max}$ and $a_{\min}$ are the maximum and minimum amplitude of the corresponding accelerometer, respectively; and K is a constant.

#### 2.2.2. PDR Position Solution

In this paper, based on the pedestrian heading angle, step size, and position information of the previous moment, the current pedestrian position is obtained:

(10) $$\left[\begin{array}{c} N_{k}\\ E_{k} \end{array}\right]=\left[\begin{array}{c} N_{k-1}+S_{k}\cdot \cos(\theta_{k})\\ N_{k-1}+S_{k}\cdot \sin(\theta_{k}) \end{array}\right]$$

where $N_{k}$ and $E_{k}$ represent the current position of the pedestrian; $N_{k-1}$ and $E_{k-1}$ represent the position of the pedestrian at the previous moment; $\theta_{k}$ represents the heading angle; and $S_{k}$ represents the step length

### 2.3. PDR/UWB Robustly Adaptive Kalman Filter Model

#### 2.3.1. Dynamic Model

PDR positioning technology calculates the pedestrian’s position based on the data collected by the acceleration sensor, gyroscope, and magnetometer, which has the characteristics of high positioning continuity and high positioning accuracy over a short time; however, the error accumulation is obvious. The UWB indoor pedestrian positioning has the characteristics of high positioning accuracy and no cumulative errors. However, gross errors exist in non-line-of-sight environments. Therefore, PDR/UWB fusion positioning can effectively improve the indoor pedestrian positioning accuracy. In this paper, the EKF algorithm is used for PDR/UWB fusion positioning. By taking the position error, distance error, and heading error as state parameters, the expression is as follows:

(11) $$X={\left[\begin{array}{cccc} dN & dE & ds & d\alpha \end{array}\right]}^{T}$$

The dynamic model expression of the PDR/UWB fusion positioning algorithm is obtained from Equation (10):

(12) $$X_{k}=FX_{k-1}+W_{k}$$

where $X_{k}$ is the system state at the current time, $X_{k-1}$ is the system state at the previous time, $W_{k}$ is the system noise vector, and F is the system state transition coefficient matrix:

(13) $$F=\left[\begin{array}{cccc} 1 & 0 & \cos\alpha_{k} & -s_{k}\times \sin\alpha_{k}\\ 0 & 1 & \sin\alpha_{k} & s_{k}\times \cos\alpha_{k}\\ 0 & 0 & 1 & 0\\ 0 & 0 & 0 & 1 \end{array}\right]$$

#### 2.3.2. Observation Model

When the UWB data are updated, the PDR/UWB fusion positioning algorithm adopts the bias of the UWB and PDR positioning results:

(14) $$Z={\left[\begin{array}{cc} \Delta N & \Delta E \end{array}\right]}^{T}={\left[\begin{array}{cc} N_{u,k}-N_{p,k} & E_{u,k}-E_{p,k} \end{array}\right]}^{T}$$

where ${\left[\begin{array}{cc} \Delta N & \Delta E \end{array}\right]}^{T}$ is the coordinate difference, $\left(\begin{array}{cc} N_{u,k} & E_{u,k} \end{array}\right)$ is the UWB positioning result, and $\left(\begin{array}{cc} N_{p,k} & E_{p,k} \end{array}\right)$ is the PDR positioning result.

However, the update frequency of PDR data is much higher than that of UWB data. Therefore, when there is no update of UWB data at the observation time of the PDR data, the following algorithm is adopted for the observation value:

(15) $$Z={\left[\begin{array}{cc} \Delta N & \Delta E \end{array}\right]}^{T}={\left[\begin{array}{cc} N_{p,k+1}^{-}-N_{p,k}^{-} & E_{p,k+1}^{-}-E_{p,k}^{-} \end{array}\right]}^{T}$$

(16) $$\{\begin{array}{l} N_{p.k}^{+}=N_{p,k}+dN\\ E_{p.k}^{+}=E_{p,k}+dE \end{array}$$

(17) $$\{\begin{array}{l} N_{k+1}^{-}=N_{k+1}^{+}+(s_{k}+ds_{k})\times \cos(\alpha_{k}+d\alpha_{k})\\ N_{k+1}^{-}=E_{k+1}^{+}+(s_{k}+ds_{k})\times \sin(\alpha_{k}+d\alpha_{k}) \end{array}$$

Formula (16) is used to update the current position of the pedestrian by using the position error obtained by filtering, and Formula (17) predicts the pedestrian’s position at the next moment by using the filter’s current time state parameters and observation values.

#### 2.3.3. PDR–UWB Fusion Positioning Model Based on EKF

The state parameter update for the PDR/UWB fusion positioning algorithm is divided into a time update and a measurement update [1], in which the time update is

(18) $$X_{k|k-1}=F_{k}X_{k-1}$$

(19) $$P_{k|k-1}=F_{k}P_{k-1}F_{k}^{T}+Q_{k-1}$$

When the observation value is obtained, the measurement is updated as follows:

(20) $$V_{k|k-1}=Z_{k}-H_{k}X_{k|k-1}$$

(21) $$P_{V_{k}}=H_{k}P_{k|k-1}H_{k}^{T}+R_{k}$$

(22) $$K=P_{k|k-1}H_{k}^{T}P_{V_{k|k-1}}^{-1}$$

(23) $$X_{k}=X_{k|k-1}+KV_{k|k-1}$$

(24) $$P_{k}=\left(I-KH_{k}\right)P_{k|k-1}{\left(I-KH_{k}\right)}^{T}+KR_{k}K^{T}$$

where $X_{k}$ and $X_{k}$, respectively, represent the optimal estimation of PDR–UWB state parameters at times k−1 and k; $P_{k-1}$ and $P_{k}$, respectively, represent the covariance matrix of the PDR–UWB state parameters at times k−1 and k; $X_{k|k-1}$ represents the predicted value of the PDR–UWB state; $Q_{k-1}$ is the system covariance matrix; $P_{k|k-1}$ is the covariance matrix of the predicted value of the state; $R_{k}$ is the covariance matrix of the observed value; $V_{k|k-1}$ is the gain factor; $P_{V_{k}}$ is the covariance matrix of the gain factor; and K is the gain matrix.

#### 2.3.4. PDR/UWB Fusion Positioning Model Based on Robustly Adaptive Kalman Filter

In order to improve the robustness and adaptability of the observation model and dynamic model of the PDR/UWB fusion positioning algorithm, a robustly adaptive filtering algorithm is established according to the positioning scene.

##### Adaptive Kalman Filter

The dynamic model accuracy of the PDR/UWB indoor positioning algorithm differs from that of the observation value model under different location scenarios. Therefore, in order to improve the adaptive fusion location algorithm in different scenarios, we dynamically adjust the covariance matrix and observation covariance matrix according to the state of the UWB data.

When there is no UWB auxiliary positioning at the observation time, the positioning accuracy of the observation value is relatively low and the prediction model accuracy is relatively high. Therefore, it is necessary to reduce the system covariance matrix to enhance the contribution rate of the state parameter prediction value for the optimal estimation, and to increase the covariance matrix of the observation value to reduce the contribution rate of the observation value with low accuracy for the optimal estimation. Through many experiments, the system covariance matrix and the observed covariance matrix are as follows:

(25) $$Q=\left[\begin{array}{cccc} 1 & 0 & 0 & 0\\ 0 & 1 & 0 & 0\\ 0 & 0 & 0.3 & 0\\ 0 & 0 & 0 & \frac{\pi }{20} \end{array}\right]$$

(26) $$R=\left[\begin{array}{cc} 10 & 0\\ 0 & 10 \end{array}\right]$$

##### Robust Kalman Filter

The accuracy of the PDR/UWB positioning model depends on the positioning accuracy of the observations when the results of the UWB positioning can be obtained at any time. Therefore, in order to reduce the influence of the UWB positioning gross error on the accuracy of the fusion positioning algorithm, a robust filtering algorithm is adopted in this paper.

Under the assumption that the observed residuals obey a Gaussian normal distribution, we construct the following test quantity [41]:

(27) $$\lambda =V_{k|k-1}P_{{}_{V_{k|k-1}}}^{-1}V_{k|k-1}{}^{T}/m$$

where m is the number of observations and λ obeys the F distribution (F(m,∞)).

According to the hypothesis test, for the given significance level α, the probability that λ is greater than $F_{\alpha }(m,\infty )$ is

(28) $$P\left(\lambda_{k}>F_{\alpha }(m,\infty )\right)<\alpha$$

In this paper, α=0.01 and m=2. When the observed value can obtain the UWB positioning result, the test factor $\lambda_{k}$ is constructed based on Equation (27). When $\lambda_{k}$ is greater than $F_{\alpha }(m,\infty )$, the covariance matrix of the observed value is dynamically adjusted by constructing the robust factor ε:

(29) $$\bar{R}=\varepsilon R$$

(30) $$\varepsilon =\frac{\lambda_{k}}{F_{\alpha }(m,\infty )}$$

**Figure 3:**
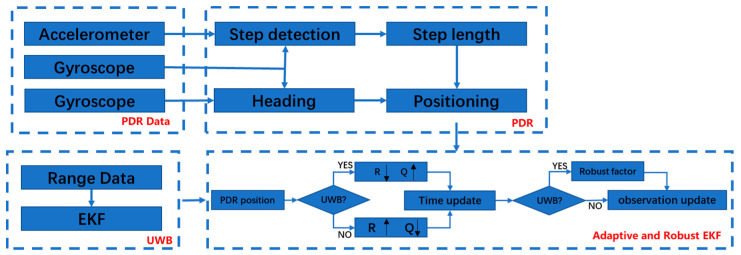
PDR–UWB fusion positioning model based on robustly adaptive Kalman filter.

Figure 3 shows the basic flow for the PDR–UWB fusion positioning algorithm. In PDR positioning technology, the data measured by accelerometers, gyroscopes, and magnetometers are used to estimate the step size and heading, and then to calculate the real-time positions of travelers. At the same time, when UWB positioning data can be observed at the observation time, the EKF algorithm is used to solve the positioning algorithm. In order to further improve the positioning accuracy of the PDR algorithm and reduce the influence of cumulative errors, the robustly adaptive EKF is used to construct the PDR/UWB fusion positioning algorithm.

### 2.4. Fusion Positioning Model with Additional Heading Constraints

**Figure 4:**
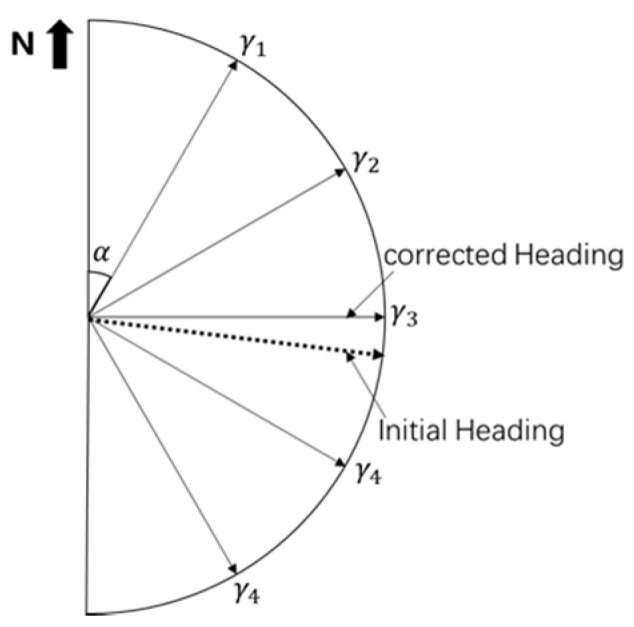
PDR heading angle correction.

As one of the important parameters of PDR, the accuracy of heading angle estimation determines the positioning performance of PDR. In order to reduce the influence of cumulative PDR errors on heading estimation, we use an indoor map to constrain the heading based on pedestrian positioning scenes:

(1) When pedestrians are in narrow passages such as corridors and stairs, the azimuth of the narrow passages is obtained according to the indoor map, and then the measured value of the pedestrian heading is corrected to the azimuth;
(2) When a pedestrian is in a wide scene, such as an indoor room, an azimuth wheel is constructed according to the pedestrian’s current position and heading. Figure 4 shows the basic principles of the course constraint based on the azimuth wheel. First, according to the pedestrian heading, the semicircular azimuth wheel is constructed at the current position and the equal angle α of the azimuth wheel is determined according to the positioning accuracy required by PDR. Then, the final heading angle $\theta_{1}$ is determined according to the relationship between the initial heading $\theta_{0}$ and the equal angle $\gamma_{i}$ of the azimuth wheel.

(31) $$\theta_{1}=\gamma_{i},if\left|\theta_{0}-\gamma_{i}\right|\leq \frac{\alpha }{2}$$

## 3. Experimental and Summary

**Figure 5:**
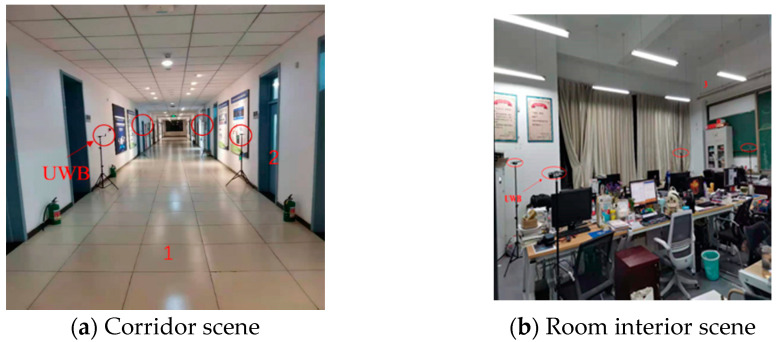
Experimental scene for the PDR/UWB fusion positioning.

The experimental scene was located on the second floor of the School of Surveying and Mapping and Urban Spatial Information of Beijing University of Civil Engineering and Architecture. First, several UWB base stations were evenly distributed in a narrow indoor corridor (the length of the corridor was 65 m, the width was 3 m, and the indoor area of the room was 6.2 m). Then, the testers carried the UWB positioning terminal and PDR equipment to perform the positioning test according to the experimental route. The specific experimental scenario is shown in Figure 5.

As shown in Figure 5, according to the different positioning environments for pedestrians, the experimental scene was divided into three parts (represented by Arabic numerals). The first part was the corridor scene, the second part was the transition scene between the corridor and the interior scene of the room, and the third part was the interior scene of the room. In the second part, because the concrete wall blocks the signal, when pedestrians are in the location scene, if the UWB base stations in the corridor and in the room are turned on at the same time, the UWB location terminal may face interference from NLOS errors.

### 3.1. Single-Sensor Positioning

#### 3.1.1. UWB Positioning

In order to demonstrate and analyze the positioning accuracy of the UWB positioning terminal in different scenarios, we first used UWB equipment for indoor positioning. At the same time, in order to verify the influence of NLOS positioning errors on positioning accuracy, all base stations were turned on at the same time at position 2 to obtain the UWB positioning error under NLOS conditions.

**Figure 6:**
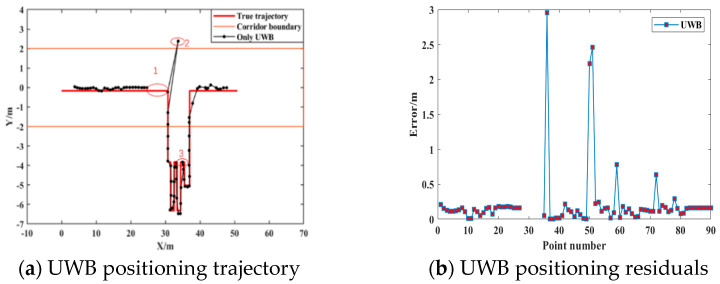
Pure UWB indoor positioning results.

Figure 6 shows the indoor positioning results for pure UWB, where Figure 6a shows the UWB positioning track and the real track and Figure 6b shows the deviation between the UWB positioning result and the real location. It can be seen from Figure 6 that when the location environment for the UWB positioning terminal was good, the indoor positioning results for UWB were in good agreement with the true track, and the positioning accuracy was better than 70 cm in general (Figure 6a). The second position was the influence of non-line-of-sight errors on the positioning results. From the track map and the residual map, it can be seen that when the observed values contained large gross errors, the UWB positioning results had serious positioning deviations, meaning the maximum error could reach 3 m (Figure 6a). The first position in the middle was used to simulate the missing UWB positioning results (for the positioning accuracy of the PDR–UWB fusion positioning algorithm in the case of missing UWB positioning results). It can be seen from position 3 in Figure 6a that when the UWB positioning base station was in the narrow space in the room, track mixing might appear because the distance between the base stations was too small.

#### 3.1.2. PDR Positioning

In order to demonstrate and analyze the positioning accuracy of the PDR indoor positioning system, we first used PDR for indoor pedestrian positioning and then obtained the positioning performance for PDR according to the real trajectory.

**Figure 7:**
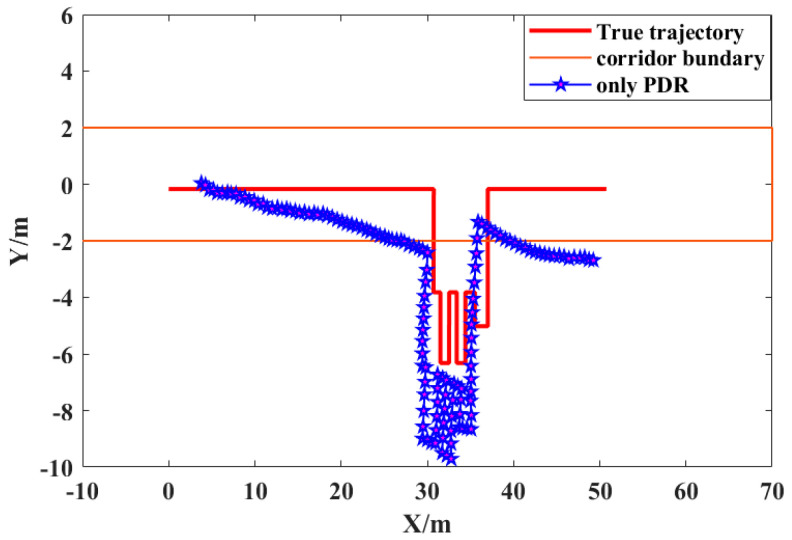
Pure PDR indoor positioning.

Figure 7 shows the indoor positioning trajectory of the PDR in detail. It can be seen from the figure that, when indoor pedestrians only relied on PDR positioning equipment to obtain their own position, the positioning results seriously deviated from the real trajectory, resulting in obvious error accumulation. At the same time, through the comparative analysis of the PDR positioning trajectory and UWB positioning trajectory, the sampling rate for the PDR positioning result was significantly better than for the UWB positioning result; however, its positioning accuracy was significantly lower than that of UWB positioning.

To sum up, when the indoor pedestrian uses UWB for positioning, when the quality of the positioning scene data is good, the accuracy of the positioning result is better than 50 cm. When there are gross errors in the observation data, the positioning result will experience serious deviation, meaning the maximum error could reach 3 m. At the same time, its data sampling rate is relatively low. When the pedestrian uses PDR for positioning, the data utilization rate is relatively high; however, the positioning result shows obvious error accumulation and the positioning error is large. Therefore, it can be seen from the above analysis that the positioning results for pure UWB indoor positioning and pure PDR indoor positioning have corresponding defects.

### 3.2. PDR/UWB Fusion Positioning

In order to demonstrate the effectiveness of the proposed algorithm, the following experiments were carried out based on the measured data:

(1) Pure UWB indoor positioning;
(2) Pure PDR indoor positioning;
(3) Indoor location of PDR/UWB based on EKF;
(4) PDR/UWB indoor positioning based on robustly adaptive EKF with heading constraint.

**Figure 8:**
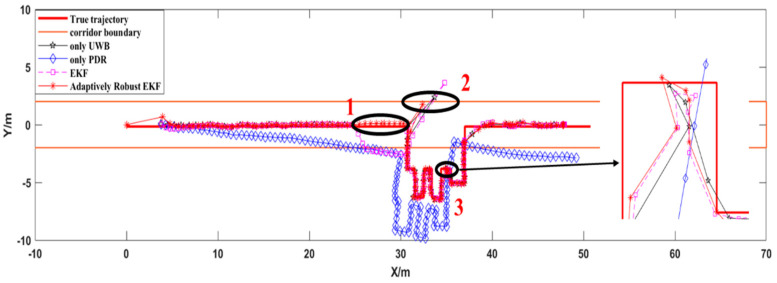
Indoor pedestrian positioning trajectory.

Figure 8 shows the positioning trajectory for each scheme and the real walking trajectory for the pedestrians. As can be seen from position 2 in Figure 8, due to the interference of non-line-of-sight error factors, the UWB observation value contained the interference of gross error factors, resulting in large deviations in the UWB positioning results. The PDR/UWB location algorithm based on conventional EKF did not reduce the weight of abnormal UWB location results, which led to large deviations in the fusion location algorithm. The PDR/UWB fusion positioning algorithm proposed in this paper effectively reduced the influence of the gross error factor on the accuracy of the fusion positioning algorithm by detecting and reducing the weight of abnormal UWB positioning results. It can be seen from position 2 that the algorithm proposed in this paper had the best resistance to gross errors.

When the UWB positioning result is missing, the accuracy of the PDR/UWB positioning fusion algorithm depends on the accuracy of the PDR positioning algorithm. It can be seen from position 1 in Figure 8 that when the conventional EKF was used for fusion positioning, the positioning result showed a large deviation due to the accuracy limit of the EKF algorithm itself and the lack of heading constraint on the PDR. In order to improve the accuracy of the fusion localization algorithm, the PDR was added with heading constraints, and the EKF system variance matrix and observation variance matrix were adaptively transformed according to the localization scene. At the same time, when the distance between the UWB base stations was small, the trajectory mixing phenomenon might appear when using the UWB positioning terminal for indoor positioning. It can be seen from position 3 in Figure 8 that the PDR/UWB fusion positioning algorithm could effectively improve the indoor positioning accuracy and reduce the trajectory mixing phenomenon.

In order to further evaluate and analyze the positioning accuracy of each algorithm, the positioning trajectory of each scheme was compared with the real trajectory and the corresponding residual positioning value was obtained.

**Figure 9:**
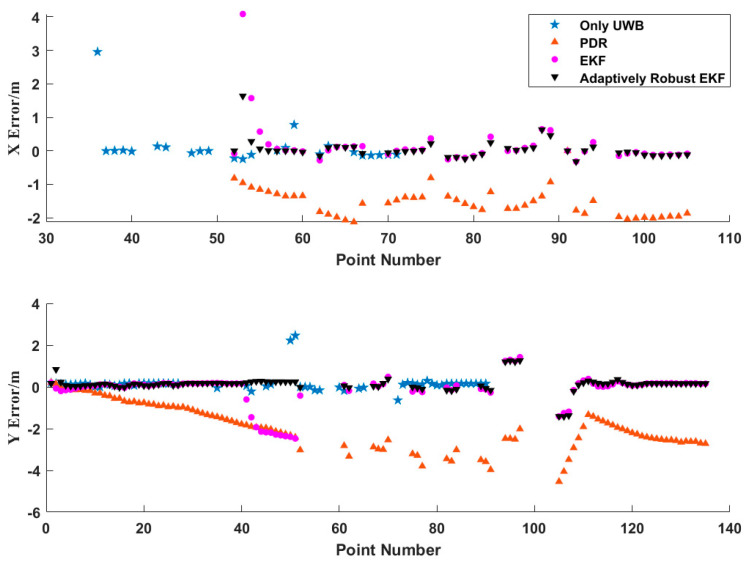
Residual positioning error of each scheme.

Figure 9 shows the positioning residuals of each scheme in the axial direction in detail. As can be seen from Figure 9, the positioning accuracy of the PDR/UWB fusion positioning algorithm based on a robustly adaptive EKF with heading constraints was optimal in all directions. Table 1 provides the root-mean-square error of the positioning error of each scheme in detail. It can be seen from Table 1 that the positioning accuracy of the proposed algorithm was better than 30 cm in the X direction, 50 cm in the Y direction, and 60 cm in the plane. Compared with the pure UWB positioning algorithm, the positioning accuracy of the proposed algorithm was improved by 32.7% in the plane and 49.8% compared with the PDR/UWB fusion positioning algorithm based on conventional EKF. At the same time, it can be seen from the figure that the positioning error of the PDR algorithm was obviously due to the error accumulation phenomenon. When the fusion positioning algorithm was used, it could effectively reduce the cumulative PDR error. Due to the limitation of the UWB adoption rate, there were relatively few data points in the UWB positioning results. The robustly adaptive EKF fusion positioning algorithm based on the heading constraint could fully maintain the excellent characteristics of the high PDR adoption rate while maintaining high positioning accuracy.

## 4. Conclusions

The UWB indoor positioning algorithm, which has a low sampling rate, is easily affected by the gross error of the observation value, and the PDR algorithm, which has a low positioning accuracy, has the defect of error accumulation. To solve these problems, we proposed a robustly adaptive EKF PDR/UWB fusion positioning algorithm based on additional heading constraints. First, the observation covariance matrix and system covariance matrix of the EKF algorithm were adaptively adjusted according to the location scene. Then, robust processing was carried out to reduce the influence of the gross error factor in the UWB positioning results on the positioning accuracy of the fusion algorithm; this method can reduce the influence of UWB observation gross errors (such as non-line-of-sight errors) on the positioning results, and it has better timeliness compared with the machine learning algorithm to identify gross errors. At the same time, in order to further improve the positioning accuracy of the algorithm, a heading constraint method based on an indoor map to constrain the heading factor of the PDR algorithm was proposed. The main advantages of this algorithm are as follows: (1) The PDR/UWB fusion localization is realized based on the improved EKF algorithm. The algorithm can automatically adjust the corresponding covariance matrix according to the UWB positioning results, which is better than the conventional EKF localization performance. (2) The existing robust theory is applied to the fusion localization algorithm. It can effectively eliminate the influence of UWB positioning errors on the fusion positioning results. (3) An improved heading constraint algorithm is proposed, which makes the algorithm better than the conventional PDR algorithm when the UWB positioning results are missing.

In order to demonstrate the effectiveness and positioning accuracy of the algorithm, experiments were carried out with the measured data. The experimental results showed that the plane positioning accuracy of our method was 0.524 m, which was 32.7% higher than that of the pure UWB positioning algorithm and 49.8% higher than that of the PDR/UWB fusion positioning algorithm based on a conventional EKF. The experimental results showed that our algorithm could effectively reduce the influence of the gross error factor on the UWB positioning results and on the accuracy of the fusion positioning algorithm and could improve the usability and accuracy of the fusion positioning algorithm in complex indoor environments. At the same time, our algorithm inherited the characteristics of high positioning accuracy from the UWB positioning algorithm and a high sampling rate from the PDR. It should be noted that the fusion localization algorithm used in this paper was an improved loose combination localization algorithm, so the authors will study the tight combination localization algorithm in the future in order to further improve the usability of the PDR/UWB localization algorithm.

## Figures and Tables

**Table 1:** RMS comparison of positioning error for different schemes.

Scheme	X	Y	Plane
Only UWB	0.633	0.453	0.778
PDR	1.582	2.104	2.632
EKF	0.681	0.792	1.045
Adaptively Robust EKF	0.294	0.434	0.524

## Data Availability

Not applicable.
